# The Complete Genome of Probiotic *Lactobacillus sakei* Derived from Plateau Yak Feces

**DOI:** 10.3390/genes11121527

**Published:** 2020-12-21

**Authors:** Kun Li, Juanjuan Liu, Zhibo Zeng, Muhammad Fakhar-e-Alam Kulyar, Yaping Wang, Aoyun Li, Zeeshan Ahmad Bhutta, Amjad Islam Aqib, Muhammad Shahzad, Jiakui Li, Desheng Qi

**Affiliations:** 1 College of Veterinary Medicine, Huazhong Agricultural University, Wuhan 430070, China; kl@mail.hzau.edu.cn (K.L.); LJJ15927022497@163.com (J.L.); zengzzhibo@163.com (Z.Z.); fakhar@webmail.hzau.edu.cn (M.F.A.K.); 15827205277@163.com (Y.W.); aoyunli1993@163.com (A.L.); 2 Institute of Traditional Chinese Veterinary Medicine, College of Veterinary Medicine, Nanjing Agricultural University, Nanjing 210095, China; 3 Department of Animal Nutrition and Feed Science, College of Animal Science and Technology, Huazhong Agricultural University, Wuhan 430070, China; qds@mail.hzau.edu.cn; 4 The Royal (Dick) School of Veterinary Studies, University of Edinburgh, Easter Bush Campus, Midlothian EH25 9RG, UK; doctorzeeshan94@gmail.com; 5 Department of Medicine Cholistan, University of Veterinary & Animal Sciences, Bahawalpur 64100, Pakistan; amjadwaseer@cuvas.edu.pk; 6 Department of Pathology, Faculty of Veterinary & Animal Sciences, The Islamia University of Bahawalpur, Bahawalpur 63100, Pakistan; m.shahzad@iub.edu.pk; 7 Laboratory of Detection and Monitoring of Highland Animal Disease, Tibet Agriculture and Animal Husbandry College, Linzhi 860000, China

**Keywords:** probiotic, yak, *Lactobacillus sakei*, genome, sequencing

## Abstract

Probiotic bacteria are receiving increased attention due to the potential benefits to their hosts. Plateau yaks have resistance against diseases and stress, which is potentially related to their inner probiotics. To uncover the potential functional genes of yak probiotics, we sequenced the whole genome of *Lactobacillus sakei* (*L. sakei*). The results showed that the genome length of *L. sakei* was 1.99 Mbp, with 1943 protein coding genes (21 rRNA, 65 tRNA, and 1 tmRNA). There were three plasmids found in this bacteria, with 88 protein coding genes. EggNOG annotation uncovered that the *L. sakei* genes were found to belong to J (translation, ribosomal structure, and biogenesis), L (replication, recombination, and repair), G (carbohydrate transport and metabolism), and K (transcription). GO annotation showed that most of the *L. sakei* genes were related to cellular processes, metabolic processes, biological regulation, localization, response to stimulus, and organization or biogenesis of cellular components. CAZy annotation found that there were 123 CAZys in the *L. sakei* genome, with glycosyl transferases and glycoside hydrolases. Our results revealed the genome characteristics of *L. sakei*, which may give insight into the future employment of this probiotic bacterium for its functional benefits.

## 1. Introduction

Probiotics are live microbes which confer health benefits to their host by supporting a healthy gut microbiota, which plays an important role in the digestive system, and the alleviation of symptoms of disease [1,2]. *Lactobacillus sakei* (*L. sakei*) is a representative probiotic that is commonly isolated from fermented foods [3]. This nonpathogenic lactic acid bacterium has been found to prevent the growth of pathogens like *Listeria monocytogenes*, *Escherichia coli* O157: H7, and *Brochothrix thermosphacta* in meat-related products [4]. Our previous study also found that *L. sakei* inhibit *Staphylococcus aureus, Pasteurella multocida, E. coli,* and *Salmonella* in vitro [5]. Alongside their antimicrobial effects, some *L. sakei* strains have been found to contribute to host health, for instance through amelioration of skin inflammation and collagen-induced arthritis [6], and through antioxidant, anti-colitis, anticancer, and anti-obesity effects [7,8,9,10]. *L. sakei* has also been found to have probiotic effects on children with atopic eczema/dermatitis [11].

Yaks are typical economically important bovine animals on the Qinghai-Tibetan Plateau [12,13]. These ruminants are resilient, in that they live and thrive in a hypoxic environment (oxygen content: 14.9~11.44%), which is cold (annual average temperature under 0~10 °C) and on a high plateau (3000~6000 m), with no guarantee of sufficient food. Their ability to adapt to such harsh environmental factors is facilitated by their large lungs, large heart, and efficient foraging and energy metabolism [14], as well as their gut microbiota which contributes significantly to live at a high altitude; also, a significant difference of microbiota was found between yaks in high and low plateaus [15,16]. The microbiota is generally recognized to play an important role in the functionality of immune systems, regulating intestinal motility, gut barrier homeostasis, and nutrient metabolism [17]. Previously, the genome of *L. sakei* has been derived from various different sources, including kimchi, meat or meat products, raw sausages, cacao beans, and potatoes, among others [3,18,19,20,21]. However, until now, there has been limited knowledge of the genome characteristics of *L. sakei* isolated from plateau animals. It is hypothesized that adaption to the critical environment of the Qinghai-Tibetan Plateau may potentially be related to gut bacteria and adapted micro-organisms, which may be profoundly influenced by the structure of their genomes. Therefore, we undertook this work to uncover the genome information of *L. sakei* isolated from high remote plateau yaks for the first time.

## 2. Materials and Methods

### 2.1. Ethics Statement

The experiments were performed under the guidance and approval of the Laboratory Animals Research Centre of Hubei, Tibet, and the ethics committee of Huazhong Agricultural University in P.R. China (Permit number 4200695757).

### 2.2. Probiotics Strain

*L. sakei* (Accession: MT712200) was isolated from the feces of a healthy female yak in Nyingchi, Tibet, China, using de Man, Rogosa and Sharp agar (Qingdao Reagents, Qingdao, China) media. It was identified by amplifying and sequencing the universal 16S ribosomal RNA gene, and comparing its sequence to the kimchi isolate from South Korea (AB650590.1) [5] in the NCBI database (https://blast.ncbi.nlm.nih.gov/Blast.cgi?PROGRAM=blastn&PAGE_TYPE=BlastSearch&LINK_LOC=blasthome). The bacterium isolate was cryopreserved at −80 °C for further analysis.

### 2.3. Genome DNA Extraction

*L. sakei* was cultured in 30 mL fresh MRS (Qingdao Reagents, Qingdao, China) medium in a 50 mL sterile tube in an incubator for 24 h at 37 °C, and the bacteria harvested by centrifugation at 8000 rpm for 40 min. The bacterial pellet was washed with sterile phosphate buffer and centrifuged at 8000 rpm for 40 min (*n* = 3). Total genomic DNA was extracted from the bacteria pellet using a QIAam 3323 Blood & Cell Culture DNA Mini Kit (QIAGEN, Hilden, Germany), following the manufacturer’s instructions. The integrity of the purified DNA was analyzed by fractionation in a 2.0% agarose gel, while DNA purity (OD260/OD280 ratio) was determined by Nanodrop 3300 Fluorospectrometer (Thermo Fisher Scientific, Wuhan, China). The DNA concentration was measured using an Invitrogen Qubit 3.0 (Thermo Fisher Scientific, Wuhan, China).

### 2.4. Library Construction and Sequencing

Extraction and purification of targeted DNA fragments was performed using a BluePippin automatic nucleic acid gel-cutting instrument (Sage Science, Beverly, MA, USA). DNA samples were fragmented, end-repaired, purified, end-linked with barcodes, purified, and linked with a sequencing adapter by employing an NBD103 and NBD114 kit (Oxford Nanopore Technologies, Oxford, UK) to construct the DNA library. The sequence library concentration was measured using an Invitrogen Qubit 3.0 (Thermo Fisher Scientific, Wuhan, China). Sequencing was carried out by utilizing a MinION Nanopore sequencer (Oxford Nanopore Technologies, Personalbio, Shanghai, China) and Illumina HiSeqTM 2000 sequencer (Personalbio, Shanghai, China).

### 2.5. Sequencing Data and Quality Control

After obtaining the Nanopore and Illumina sequence of the generated raw data, quality control was performed by using the Quality Score and content distribution. The base *Q*-Score is a commonly used evaluation parameter to represent reliability and accuracy. The base content distribution was analyzed to detect the separation of AT and GC. Reads with connectors and low-grade reads (proportion of N > 10%, over half of a read *Q*-Score ≤ 10) were removed to ensure high quality and accuracy for the subsequent analysis.

### 2.6. Genome Assembly, Annotation, and Phylogenetic Analysis

The SPAdes genome assembler (v3.11.1) and A5-miseq (v20160825) were used to assemble sequencing data generated by Illumina [22,23]. HGAP4 and CANU (V1.6) were utilized to assemble sequencing data generated by Nanopore [24,25]. Then, MUMmer (v4) was employed to retrieve previously assembled results [26], while pilon (v1.22) was used to correct presently assembled results [27]. The annotation of the *L. sakei* genome was performed via GeneMarkS (v4.32) for prediction of protein-coding genes [28]; tRNAscan-SE (v1.3.1) for prediction of tRNA [29]; and Barrnap (0.9-dev) (https://github.com/tseemann/barrnap) for prediction of rRNA. All other RNAs were predicted by blasting with the Rfam database [30]. RepeatMasker (v4.0.3) (http://www.repeatmasker.org/) was used for analyzing repetitive sequences. A phylogenetic tree was constructed based on 16S rDNA gene sequences from available Lactobacillus strains, including *L. sakei* (MT814885.1, MK640920.1, MF688992.1, MF688992.1, KP179418.1, KM267631.1, KF559316.1, HQ992696.1, HQ022862.1, EU755262.1), *L. johnsonii* (M99704.1, NR_117574.1), *L. paralimentarius* (NR_043096.1), *L. ultunensis* (NR_042802.1), *L. harbinensis* (NR_041263.1), *L. hominis* (NR_125548.1), *L. acetotolerans* (M58801.1), *L. reuteri* (MT764998.1), and *Pediococcus damnosus* (D87678.1). A whole genome-based phylogenetic tree was constructed based on available Lactobacillus strains, including *L. sakei* (CP032633, CP046037, LT960781, LT107933, NZ_CP05969, NZ_LT960788, NZ_LP022709), and *P. damnosus* (CP012294).

### 2.7. Functional Analysis

All the protein-coding genes were blasted in the database of Nr (ftp://ftp.ncbi.nih.gov/blast/db/), Carbohydrate-Active enZYmes Database (http://www.cazy.org), Swiss-Prot (http://www.uniprot.org/), GO (http://www.geneontology.org/), eggNOG/COG (http://www.ncbi.nlm.nih.gov/COG/), KOG (ftp://ftp.ncbi.nih.gov/pub/COG/KOG/kyva), KEGG (http://www.genome.jp/kegg/), and Pfam (http://pfam.xfam.org/), respectively, to perform gene function analysis of *L. sakei’s* genome. KEGG pathway annotation was performed using KAAS (Ver. 2.1) [31].

### 2.8. Plasmid Analysis

The annotations of *L. sakei* plasmids and protein-coding genes were blasted in the databases similar to those used for genome analysis.

### 2.9. Genome and Plasmid Comparison

In order to determine the genomic structure and plasmids of *L. sakei*, the genome sequence data was analyzed in comparison to the NCBI genome database (https://www.ncbi.nlm.nih.gov/genome/?term=Lactobacillus+sakei).

## 3. Results

### 3.1. Raw Data Deposit

The sequences of *L. sakei’s* genome and plasmids were submitted to the NCBI database with the accession numbers: chromosome (CP064817) and plasmids (MW265923, MW265924, MW265925).

### 3.2. Sequencing Information and Quality Control Results

**Figure 1:**
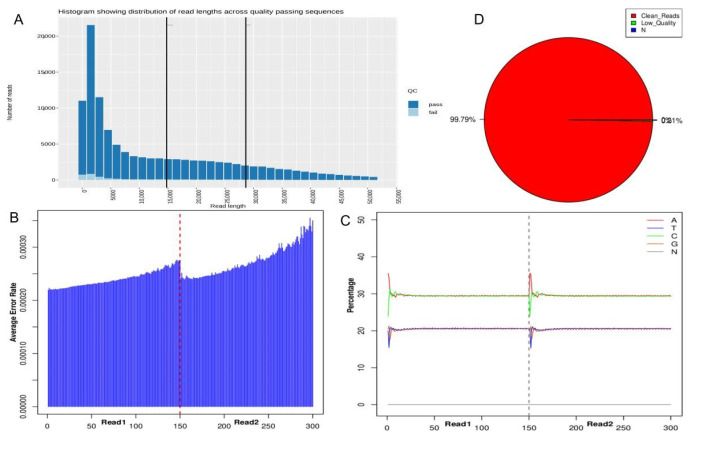
Sequencing quality control information of *Lactobacillus sakei (L. sakei). (***A**) Histogram of reading lengths and number distribution of sequencing; (**B**) Base sequencing error rate distribution map; (**C**) Bases (ATGC) content distribution map; (**D**) Original data composition map.

A total of 113,051 filtered reads (96.29%), with a total of 1,671,594,841 bp, were obtained through Nanopore sequencing (Table 1). Among these, low-quality reads were less than 5000 (Figure 1A). Illumina sequencing showed that all the read error rates were less than 0.00035 (Figure 1B). The distribution of A/T/C/G contents were all nearly horizontal, except in the first few reads (presented as peaks) (Figure 1C), because of preference for random primer sequences in the initial reads. Overall, the clean reads of *L. sakei* were as high as 99.79% (7074292/7089390), with a total base of 1,061,058,974 bp (Table 2, Figure 1D). The Q-Scores of Q20 and Q30 were 97.95% and 93.42%, respectively (Table 2), revealing the high quality and accuracy of the *L. sakei* genome sequence reads.

### 3.3. Genome Assembly, Annotation and Phylogenetic Tree

**Figure 2:**
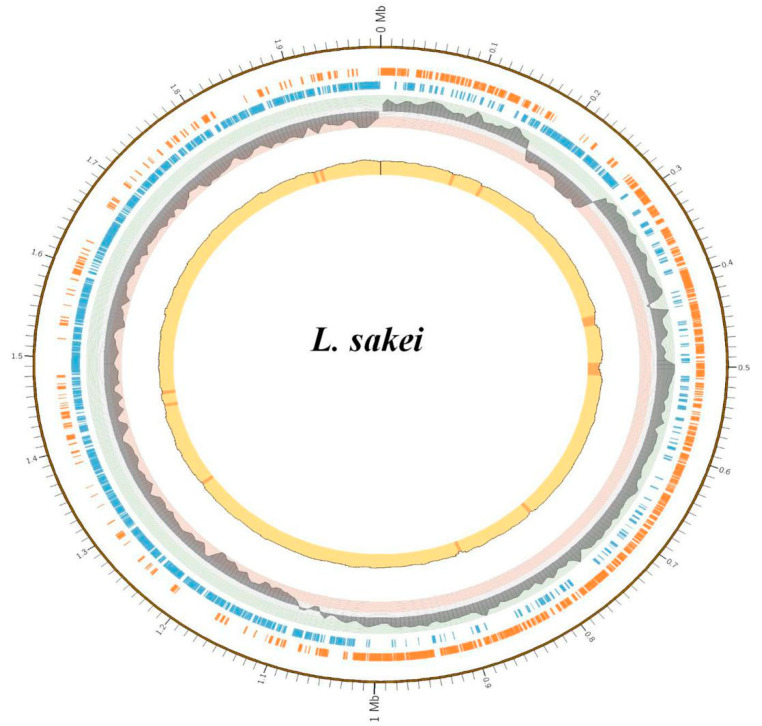
The genome circular map of *L. sakei*.

**Figure 3:**
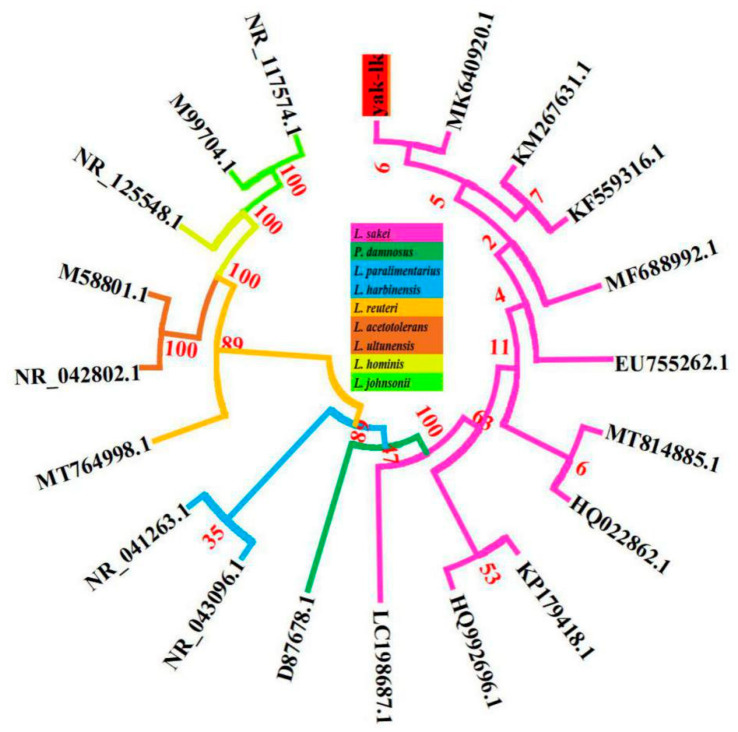
Phylogenetic tree of the *L. sakei* yak isolate and references strains. The tree was constructed using software MEGA 6.0 by the neighbor-joining method, based on 16S rDNA gene sequences with 1000 replications in bootstrap testing.

A total of 1,989,121 bp (1.99 Mbp) were sequenced from *L. sakei’s* circular genome, with a GC content of 41.1%. The sequencing depth of *L. sakei* was 533, which could reduce false positives and sequencing error rates. Prodigal-2.6.2 (https://github.com/hyattpd/prodigal/wiki) was used to perform the gene prediction of *L. sakei*, and the results showed that the total gene length was 1,776,396 bp with an average gene length of 875 bp (Table 3). The GC content of *L. sakei* genes was observed to be 41%, with an intergenetic region length of 212,724. Gene density and Gene/Genome (%) were 1045 and 89%, respectively. In total, *L. sakei* had 1943 protein coding genes, 21 rRNA (equal number of 5S rRNA, 16S rRNA, 23S rRNA), 65 tRNA, and 1 tmRNA. A total of 103 repeated sequences were observed, in which 49 sequences were located in rRNA, 45 were simple repeated sequences, and 9 were low-complexity repeat sequences. The *L. sakei* genome map was drawn using cgview (Figure 2) [32]. The 16s rRNA phylogenetic tree showed that the *L. sakei* yak isolate is the closest evolutionary relative of *L. sakei* (MK640920.1) (Figure 3). The whole genome-based phylogenetic tree showed that the *L. sakei* yak isolate is the closest evolutionary relative of *L. sakei* (NZ_CP059697) (Figure S1).

### 3.4. Functional Analysis

**Figure 4:**
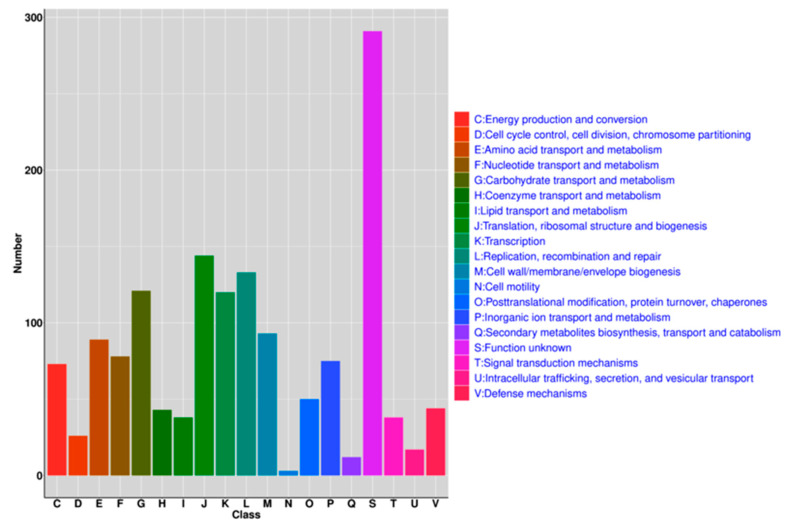
The eggNOG annotation of the *L. sakei* genome.

**Figure 5:**
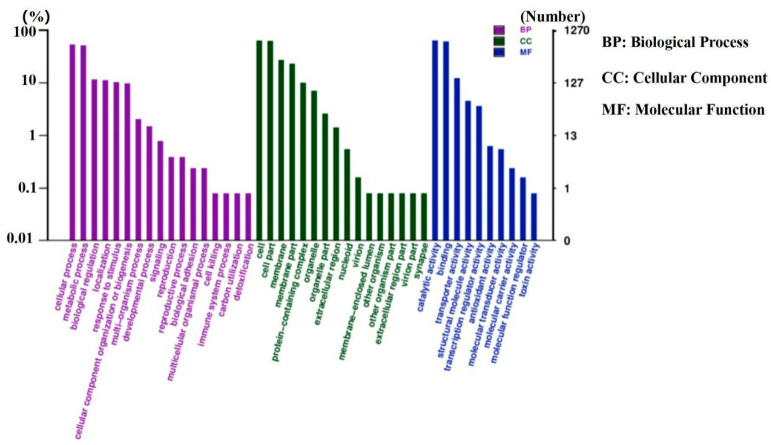
The GO annotation of the *L. sakei* genome.

EggNOG annotation uncovered that the mainly known genes of *L. sakei* genes were found to belong to J (translation, ribosomal structure, and biogenesis), L (replication, recombination, and repair), G (carbohydrate transport and metabolism), and K (transcription) (Figure 4). GO annotation showed that most of the *L. sakei* genes are related to biological processes, metabolic processes, biological regulation, localization, response to stimuli, and cellular component organization or biogenesis. In terms of function, most genes contribute to cell structural functions, including membrane and organelle architecture. In terms of cell molecular functions, the majority of genes were related to enzymatic catalytic activity, ligand, and transporting activities (Figure 5).

**Figure 6:**
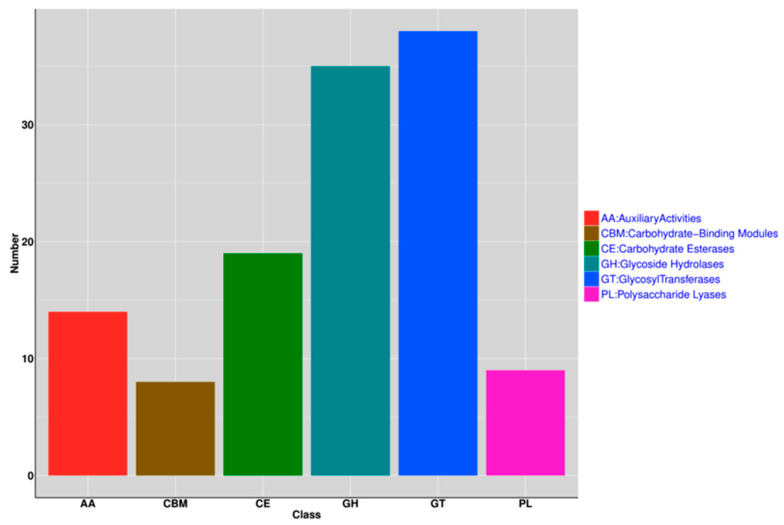
Analysis of CAZy in the *L. sakei* genome.

CAZy annotation found that there are 123 CAZys in the *L. sakei* genome, including glycosyl transferases and glycoside hydrolase (Figure 6). Signal peptides were analyzed via SignalP (v4.1), with 24.61% (493/2003) coding genes containing signal peptides [33]. Transmembrane domain prediction performed by employing TMHMM (Server v2.0) found that 28.61% (573/2003) of coding genes have transmembrane domains [34]. Sequence blasting of genes with the NCBI database, and protein-coding via diamond (v0.9.10.111), indicated 99.70% (1997/2003) of genes were protein-coding genes [35] while blasting with the Swiss-Prot database showed 65.60% (1314/2003) of the gene code for proteins. Pfam (http://pfam.janelia.org/) blasting revealed that 72.89% (1460/2003) of the encoded proteins contain PFAM domains [36].

**Figure 7:**
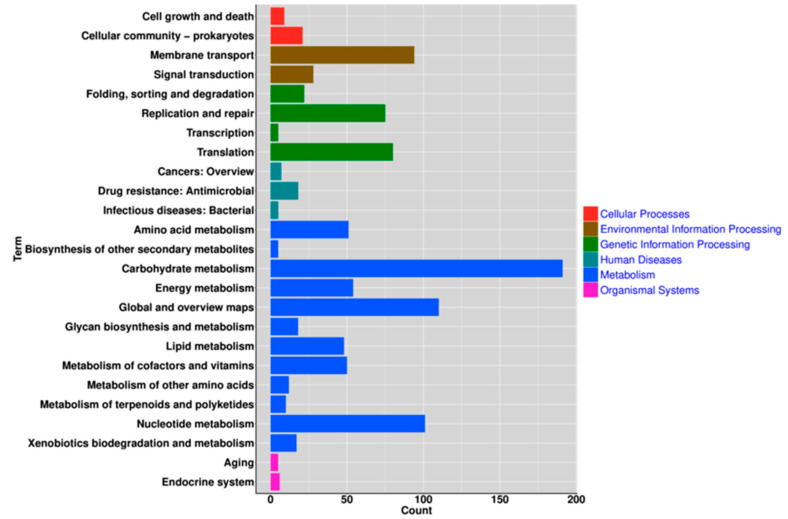
The KEGG annotation of the *L. sakei* genome.

KEGG annotation found that the biosynthesis of secondary metabolites, the metabolism of amino acids, carbohydrates, energy, glycans, lipids, cofactors, vitamins, terpenoids, polyketides, and nucleotides, and xenobiotic metabolism and biodegradation, were the dominant functions of the genes (Figure 7). KEGG pathways analysis revealed 72 “ko-” related reference pathways of metabolism (glycolysis/gluconeogenesis, pentose phosphate, pentose and glucuronate interconversions, fructose and mannose, galactose, purine, pyrimidine, amino acids, alanine, aspartate, glutamate, glycine, serine, threonine, cysteine, methionine and tyrosine, selenocompound, glutathione, starch, sucrose, amino sugar and nucleotide sugar, glycerophospholipid, pyruvate, glycerolipid, glyoxylate and dicarboxylate, propanoate, butanoate, one carbon pool by folate, methane, thiamine, nicotinate and nicotinamide, biotin, drug, carbon, fatty acids); biosynthesis (fatty acids, amino acids, phenylalanine, streptomycin, peptidoglycan, pantothenate and CoA, folate, terpenoid backbone, aminoacyl-tRNA); degradation (fatty acids, chloroalkane and chloroalkene, naphthalene, aromatic compounds, ribosomes, RNA), and other pathways, like oxidative phosphorylation, photosynthesis, carbon fixation in photosynthetic organisms, carbon fixation pathways in prokaryotes, β-Lactam resistance, cationic antimicrobial peptide (camp) resistance, ABC transporters, two-component system, quorum sensing, phosphotransferase system (PTS), RNA polymerase, RNA replication, protein export, bacterial secretion system, base excision repair, nucleotide excision repair, mismatch repair, homologous recombination, cell cycle-caulobacter, staphylococcus aureus infection, longevity regulating pathway-worm, glucagon signaling pathway, and central carbon metabolism in cancer.

### 3.5. Plasmid Analysis

**Figure 8:**
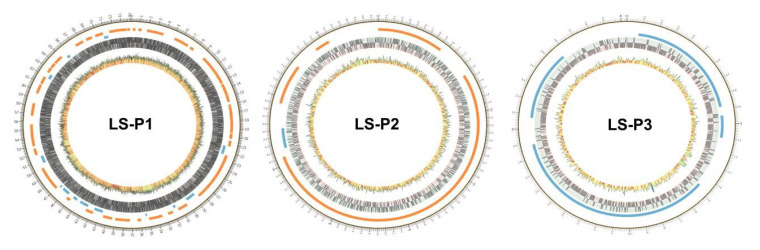
The genome circular maps of *L. sakei* plasmids.

There were three circular plasmids (LS-P1, LS-P2, LS-P3) in *L. sakei* (Figure 8). The gene length of those three plasmids was 74,183, 8784 and 4034 bp, respectively. The GC content in LS-P1 was 40%, while a slightly lower GC content was found in LP-P2 and LS-P3 (35%; 35%). Prokka (v1.12) was used to annotate the *L. sakei* plasmids [37]; LS-P1 encoded for 75 proteins, including Lactococcin A secretion protein LcnD, IS3 family transposase IS1163, and Asparagine synthetase B. LS-P2 and LS-P3 encoded for 7 and 6 hypothetical proteins, respectively.

### 3.6. Genome and Plasmid Comparison Analysis

The genome length (1.99 Mbp), GC content (41.1%), coding genes (1943), and plasmids (*n* = 3) of yak *L. sakei* were compared to other *L. sakei* strains’ genomes in the databases. About 54 of the previously reported strains had comparable genome length (1.82~2.19 Mbp), GC content (40.6~41.3%), coding genes (1752~3532), and plasmids (0~3) (Table 4). On the other hand, 42 of the available *L. sakei* genomes had divergent plasmid length (1526~84,581), GC content (34.0~44.5%), and coding genes (2~80) (Table 5).

## 4. Discussion

In the current study, we performed genome sequencing of *L. sakei* via both the Nanopore and Illumina sequencing platforms. The genome size, GC content, and number of coding proteins (1943) of the yak *L. sakei* isolates were compared to previously reported strains (Table 4) [3,18,19,38,39,40]. The number of rRNA genes (22) of *L. sakei* was higher than most of the previously reported isolates (9~21). Interestingly, most of the previously reported *L. sakei* strains had 0~2 plasmids, with lengths ranging from 1526 bp to 84,581 bp, while the present yak isolates had three plasmids, with lengths of 4034~74,183 bp (Table 5).

A total of 49 repeated RNA genes were found in the *L. sakei* genome, with a high redundancy, which may promote the bacterium ability to flourish in a complex microbial environment (Figure 2) [40]. In the KEGG annotation, we found that the *L. sakei* genome was rich in energy metabolism-related genes (Figure 7). In the 72 “ko-” signaling pathways, carbohydrate-like glycolysis (ko00010), pentose phosphate (ko00030), pentose and glucuronate interconversions (ko00040), fructose and mannose metabolism (ko00051), galactose metabolism (ko00052), starch and sucrose metabolism (ko00500), amino sugar and nucleotide sugar metabolism (ko00520), and pyruvate metabolism (ko00620) were found. Lipid pathways, such as fatty acid biosynthesis (ko00061), fatty acid degradation (ko00071), glycerolipid metabolism (ko00561), glycerophospholipid metabolism (ko00564), glyoxylate and dicarboxylate metabolism (ko00630), propanoate metabolism (ko00640), butanoate metabolism (ko00650), and fatty acid metabolism (ko01212) were uncovered. ATP-related oxidative phosphorylation (ko00190) and phosphotransferase system (PTS) (ko02060), with essential carbohydrate and lipid signaling pathways, may play an important role in promoting host energy storage and metabolism, especially in harsh conditions. PTS is comprised of two cytoplasmic phosphoryl proteins and sugar-specific enzyme II complexes, which have many regulatory functions in metabolism (carbon, nitrogen, and phosphate), transport (chemotaxis and potassium), and pathogen virulence [41,42]. Bacteria using PTS to transport carbohydrates into the bacterial cells contribute to metabolism [43]. These results add to the evidence for specific adaptation characteristics of the *L. sakei* yak strain. Interestingly, besides β-lactam resistance (ko01501) and cationic antimicrobial peptide resistance (ko01530) pathways, antibacterial signaling, e.g., Streptomycin (ko00521) during *Staphylococcus aureus* infection (ko05150) may keep yaks from being infected by specific pathogens, as *Staphylococcus aureus* can modulate the sensitivity to cationic antimicrobial peptides to decrease the immune response (https://www.kegg.jp/dbget-bin/www_bget?ko05150) [44]. Our previous study also confirmed that this *L. sakei* strain was not only resistant to commonly used antibiotics by letting them survive during the treatment of *Staphylococcus aureus* infection, but also has strong antibacterial ability to *Staphylococcus aureus* in vitro [5]. Other pathways like longevity regulating pathway-worm (https://www.kegg.jp/dbget-bin/www_bget?ko04922), glucagon signaling pathway (https://www.kegg.jp/dbget-bin/www_bget?ko04922), and central carbon metabolism in cancer (https://www.kegg.jp/dbget-bin/www_bget?ko05230) were signaling related to glucose metabolisms and mitochondrial respiration, which may indicate the probiotic characteristics of this bacterium. However, further study is needed to evaluate such signaling.

The gut microbiota genome (CAZys encoded) contributes significantly to host carbohydrate disintegration, which has been found to be indispensable in human nutrition [45]. CAZy annotation uncovered that 35 and 38 genes encode glycosyl transferases (GTs) and glycoside hydrolases (GHs) in the *L. sakei* genome, respectively (Figure 6). Key enzyme genes of GTs of *L. sakei* were in line with those in *L. pentosus* (29), while GHs were considerably higher than in *L. pentosus* (10) [45]. These GHs are necessary for glycosidic bond hydrolysis. Its richness in yak strains may be beneficial for carbohydrate metabolism.

## 5. Conclusions

This study provides an overview of the genome and reports on unique genetic properties of probiotic *L. sakei* isolated from yak feces. Genomic analysis can determine the properties of this strain and may show the application of new biotechnology to key strains in the industry. However, further studies of the molecular and physiological properties of released metabolic products are required in order to understand the nature of the association of these probiotics with its metabolites. We hope that future research will help in understanding the biology of metabolites better, and provide new insights into the interactions between metabolites and bacteria.

## Figures and Tables

**Table 1:** Sequencing information of *L. sakei* via Nanopore.

Reads Type	Bases (bp)	Reads Number	Reads Mean Length (bp)	Reads N50 (bp)	Longest Reads (bp)	*Q*-Score
Raw	1,722,592,614	117,399	14,672.97	28,556	179,164	
Filtered	1,671,594,841	113,051	14,786.2	28,642	179,164	≥7

**Table 2:** Sequencing information of *L. sakei* via Illumina.

Total Reads	Clean Reads	Percentage (%)	Clean Bases	GC Content (%)	Q20 (%)	Q30 (%)
7,089,390	7,074,292	99.79%	1,061,058,974	41.0%	97.95%	93.42%

**Table 3:** Statistical results of the gene prediction of *L. sakei.*

Category	Property
Total gene length (bp)	1,776,396
Average gene length (bp)	875
GC content in the gene region	41
Gene density(genes/Mb)	1045
Gene/Genome (%)	89
Intergenetic region length	212,724
Protein coding genes	1943
rRNA genes	21
tRNA genes	65
Gene density(genes/Mb)	1045
Gene/Genome(%)	89
Intergenetic region length	212,724
Sequencing depth	533

**Table 4:** Characteristics’ analysis of available *L. sakei* genome.

Accession No.	Size (Mbp)	GC (%)	Genes No.	Plasmids No.	Origin
CP064817	1.99	41.1	1943	3	Yak feces
NZ_CP046037.1	2.02	41.1	1904	2	Kimchi
NZ_CP025839.1	2.10	41.1	1964	0	Kimchi
NZ_CP048116.1	1.99	41.0	1842	0	Kimchi
NZ_CP025136.1	2.03	41.2	1936	2	Kimchi
NZ_CP025206.1	2.04	41.0	1916	2	Kimchi
NZ_CP020806.1	2.08	41.2	1975	2	Kimchi
NZ_CP025203.1	2.04	41.2	1913	2	Kimchi
JRFY01000024.1	2.19	40.6	1869	~	Kimchi
NZ_LT960781.1	2.10	41.1	2024	2	Sausage
NZ_LT960790.1	2.01	41.3	1886	2	Sausage
NZ_LT907930.1	1.82	41.0	3532	0	Sausage
NZ_LT907929.1	1.88	41.1	1752	0	Sausage
NZ_ASTI01000039.1	2.03	40.9	1919	1	Sausage
NZ_CP059697.1	2.06	41.0	2002	1	Fermented vegetables
NZ_CP032652.1	2.02	41.2	1908	1	Fermented vegetables
NZ_CP032640.1	2.02	41.2	1907	1	Fermented vegetables
NZ_MKDM00000000.1	2.02	40.9	1934	~	Meat
NZ_MKDO00000000.1	2.03	40.9	1938	~	Meat
NZ_MKDC00000000.1	1.94	41.1	1892	~	Meat
NZ_MKDN00000000.1	1.91	41.0	1838	~	Meat
NZ_AZFG01000049.1	1.99	41.0	1923	~	Meat
NZ_LT907933.1	1.98	41.0	1864	0	Horse meat
NZ_LT960788.1	2.04	41.1	1936	1	Beef carpaccio
NZ_LT960784.1	2.06	41.1	1928	3	Beef carpaccio
NZ_CP022709.1	2.08	41.2	1955	2	Water
NZ_LSFF00000000.1	2.01	41.0	1929	~	Mukeunji
NZ_CP032633.1	2.02	41.2	1909	1	Raw cow milk
NZ_CP032635.1	2.02	41.2	1909	1	Raw cow milk
NZ_AP017931.1	1.99	41.2	1898	3	Microbial mat material
NZ_CP020459.1	2.06	41.0	1921	2	Food
NZ_LT960777.1	1.96	41.2	1813	3	Human feces
NZ_CABMJT000000000.1	1.96	41.2	1813	~	Human gut
NZ_OVTU00000000.1	2.06	40.8	1986	~	Potatoes
NZ_OKRC00000000.1	2.03	40.9	1972	~	Potatoes
NZ_CABFKU000000000.1	2.00	41.0	1923	~	Human nasopharynx
NZ_SCIF00000000.1	1.98	41.0	1915	~	Bovine
NZ_QOSE00000000.1	1.94	41.1	1859	3	Sauerkraut
NZ_MKGH01000040.1	1.95	41.0	1878	~	Cacao bean fermentation
NZ_MKGG01000009.1	1.91	41.0	1847	~	Cacao bean fermentation
NZ_BJVN01000001.1	1.90	41.1	1822	1	Moto starter of sake
NZ_AP017929.1	1.94	41.2	1834	1	Moto starter of sake
NZ_AZDN01000001.1	1.91	41.0	1818	~	Moto starter of sake
NZ_MKDI01000019.1	1.93	41.0	1849	~	~
NZ_BKAA01000001.1	1.97	41.0	1920	~	~
NC_007576.1/CR936503.1	1.88	41.3	1777	0	~
BALW01000001.1	1.91	41.0	~	~	~
NZ_PUFE01000027.1	1.94	41.1	1838	~	~
NZ_LT907931.1	1.90	41.1	1782	0	~
NZ_MKGB01000012.1	1.99	41.0	1900	~	~
NZ_MKDL01000023.1	1.97	40.9	1890	~	~
NZ_MKDJ00000000.1	1.92	41.1	1848	~	~
NZ_MKDD00000000.1	2.01	41.0	1926	~	~
NZ_BJLN00000000.1	1.94	41.1	1855	~	~
NZ_MKDE00000000.1	1.95	40.9	1885	~	~

~ = Unavailable.

**Table 5:** Characteristics’ analysis of *L. sakei* plasmids.

Accession No.	Size (bp)	GC (%)	Genes No.	Origin
MW265923	74,183	40.0	75	Yak feces
MW265924	8784	35.0	7	Yak feces
MW265925	4034	35.0	6	Yak feces
NZ_ASTI01000039.1	20,510	37.6	17	Sausage
NZ_LT960791.1	33,463	41.2	28	Sausage
NZ_LT960792.1	13,205	43.5	14	Sausage
NZ_LT960782.1	46,347	39.2	43	Sausage
NZ_LT960783.1	1526	40.2	2	Sausage
NZ_CP020460.1	84,581	34.5	80	Food
NZ_CP020461.1	26,098	40.4	21	Food
NZ_CP022710.1	75,468	41.5	57	Water
NZ_CP022711.1	11,504	34.6	10	Water
NZ_AP017932.1	33,266	40.0	32	Microbial mat material
NZ_AP017933.1	6196	35.9	6	Microbial mat material
NZ_AP017934.1	4315	35.3	5	Microbial mat material
NZ_LT960778.1	31,701	37.3	27	Human feces
NZ_LT960779.1	12,663	43.4	13	Human feces
NZ_LT960780.1	11,068	39.3	9	Human feces
NZ_LT960789.1	47,094	40.7	38	Beef carpaccio
NZ_LT960786.1	11,996	44.5	13	Beef carpaccio
NZ_LT960787.1	11,156	39.5	8	Beef carpaccio
NZ_LT960785.1	57,338	38.8	50	Beef carpaccio
NZ_CP046038.1	53,648	39.5	53	Kimchi
NZ_CP046039.1	20,312	38.5	21	Kimchi
NZ_CP020807.1	13,019	41.2	9	Kimchi
NZ_CP020808.1	13,083	38.1	10	Kimchi
NZ_CP025137.1	42,297	41.4	32	Kimchi
NZ_CP025138.1	14,883	36.3	20	Kimchi
NZ_CP025207.1	45,431	40.4	42	Kimchi
NZ_CP025208.1	44,839	38.5	38	Kimchi
NZ_CP025204.1	75,290	41.1	66	Kimchi
NZ_CP025205.1	14,876	36.3	20	Kimchi
NZ_CM010625.1	11,246	39.7	8	Sauerkraut
NZ_CM010626.1	6593	35.9	8	Sauerkraut
NZ_CM010627.1	2499	33.8	3	Sauerkraut
NZ_CP032636.1	51,286	41.9	40	Raw cow milk
NZ_CP032634.1	51,286	41.9	42	Raw cow milk
NZ_CP032641.1	51,284	41.9	39	Fermented vegetables
NZ_CP032653.1	51,286	41.9	39	Fermented vegetables
NZ_AP017930.1	6214	36.0	7	Moto starter of sake
NZ_CP059698.1	56,165	41.1	48	~
NC_004942.1	12,959	38.1	13	~
EF605268.1	5031	36.7	3	~
EU555173.1	1790	34.0	2	~

~ = Unavailable.

## Supplementary Materials

The following are available online at https://www.mdpi.com/2073-4425/11/12/1527/s1, Figure S1: Phylogenetic tree of the *L. sakei* yak isolate and references strains. The tree was constructed using software MEGA 6.0 by the neighbor-joining method, based on whole genome sequences with 1000 replications in bootstrap testing.
