# Supplementary material for: The Complete Genome of Probiotic Lactobacillus sakei Derived from Plateau Yak Feces
Source: Genes (Basel). 2020 Dec 21;11(12):1527. doi: 10.3390/genes11121527 (PMC7766009; doi:10.3390/genes11121527)
Supplement: Supplementary file 1 [file genes-11-01527-s001.pdf]

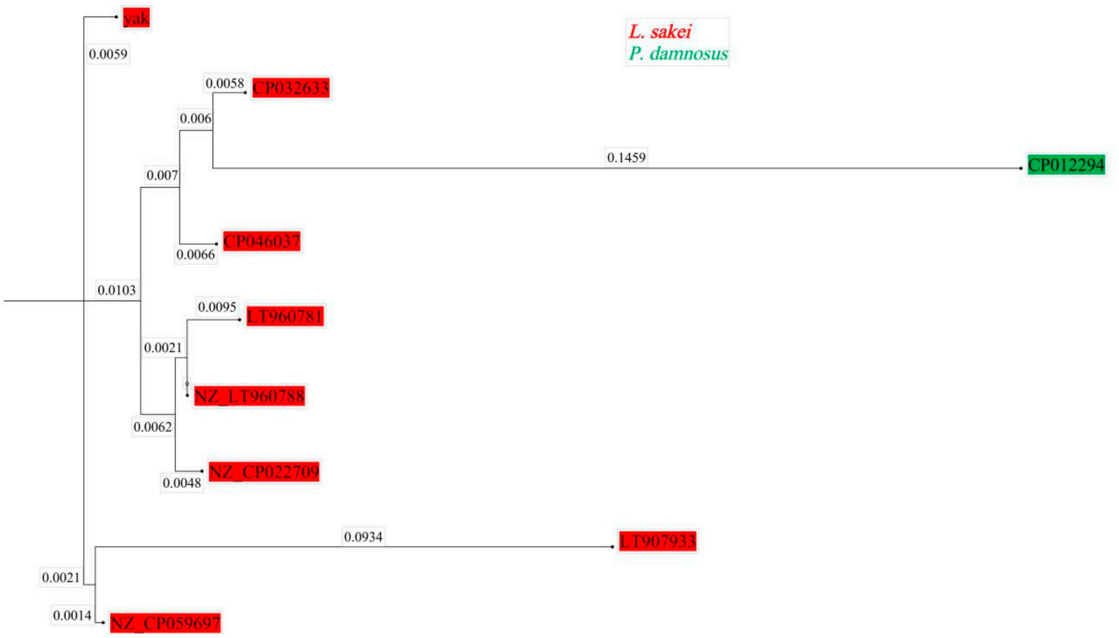

Figure S1. Phylogenetic tree of the *L. sakei* yak isolate and references strains. The tree was constructed using software MEGA 6.0 by the neighbor-joining method, based on whole genome sequences with 1000 replications in bootstrap testing.
